# Willingness to Pay for Blood Pressure Self‐Monitoring in People With Prehypertension

**DOI:** 10.1111/jch.70247

**Published:** 2026-04-11

**Authors:** Valerio Benedetto, Andrew J. Clegg, Emma P. Bray, Paul Heyworth, Lucy Hives, Joseph Spencer, Caroline Leigh Watkins, Nefyn Williams, the REVERSE study group

**Affiliations:** ^1^ Research Fellow, Applied Health Research hub University of Lancashire Preston UK; ^2^ NIHR Applied Research Collaboration North West Coast Liverpool UK; ^3^ Professor of Health Services Research Applied Health Research hub University of Lancashire Preston UK; ^4^ Senior Research Fellow, Stroke Research Team School of Nursing and Midwifery University of Lancashire Preston UK; ^5^ No affiliation, Patient representative Lancashire UK; ^6^ Research Associate, School of Nursing and Midwifery University of Lancashire Preston UK; ^7^ Research Assistant, Clinical Trials Research Unit University of Sheffield Sheffield UK; ^8^ Professor of Stroke and Older People's Care, Stroke Research Team School of Nursing and Midwifery University of Lancashire Preston UK; ^9^ Applied Health Research hub University of Lancashire Preston UK; ^10^ Professor in Primary Care Department of Primary Care and Mental Health University of Liverpool Liverpool UK

**Keywords:** blood pressure, general practice, prehypertension, self‐monitoring, willingness to pay

## Abstract

Prehypertension is defined as blood pressure (BP) between 120–139/80–89 mmHg. It affects around 40% of adults, increasing their risk of developing hypertension and cardiovascular disease‐related conditions.

The “Risk rEduction interVEntion for Raised blood preSsurE” (REVERSE) feasibility study investigated whether self‐monitoring of BP was acceptable and feasible for managing prehypertension. As part of REVERSE, a willingness‐to‐pay (WTP) analysis assessed the economic value of the BP self‐monitoring intervention.

A WTP questionnaire asked participants how much and why they would be willing to pay for a BP machine and for additional support and training around BP self‐monitoring. The associations between the total WTP amount (for the BP machine plus the additional support and training) and participants’ sociodemographic and clinical characteristics were investigated using generalized linear modelling (GLM).

WTP data was collected from 66 participants (median age: 58.50 years; females: 59.09%). Most of the participants (72.73%) lived in areas of low socioeconomic deprivation. The median total WTP amount was £41.37 in 2024 prices (interquartile range: £36.20–£93.09). The BP machine functions/facilities, the amount reflecting potential benefit, and being a reasonable value were the most cited reasons behind the valuations.

The GLM regression showed that higher WTP values were associated with the functions/facilities and potential benefit of the BP machine.

We believe this to be the first study to provide insights around the economic value of BP self‐monitoring in prehypertension. Further research based on larger, and more representative, samples is needed to validate these findings.

**Trial registration number**: ISRCTN13649483

## 1 Introduction

Prehypertension is defined as having blood pressure (BP) in the range between 120–139/80–89 mmHg [1], with estimates indicating that approximately 40% of adults have their BP within that range [2]. Prehypertension is not classed as a disease but is associated with a risk of developing hypertension [3] and cardiovascular disease (CVD) [4]. In the United Kingdom, diseases caused by hypertension cost the NHS over £2 billion a year [5]. To manage prehypertension both clinical guidance [6] and evidence [7] highlight the effectiveness of non‐pharmacological interventions focusing on lifestyle modifications.

Self‐monitoring interventions have been shown to be effective in reducing the risk of developing CVD in people with hypertension [8] and diabetes [9]. Whether self‐monitoring could benefit people with prehypertension remains uncertain, in particular whether individuals will engage with the concept of prehypertension, perceive it as a risk, and be willing to intervene at BPs at this level [10, 11, 12, 13].

The “Risk rEduction interVEntion for Raised blood preSsurE” (REVERSE) feasibility study [14] aimed to tackle this uncertainty by determining the feasibility and acceptability of BP self‐monitoring amongst people with prehypertension, and healthcare professionals (HCPs). The results from the REVERSE study will inform the design of a multi‐center, randomized controlled trial which is expected to evaluate the effectiveness of self‐monitoring in detecting and increasing awareness of prehypertension. One of the objectives of the REVERSE study was to evaluate the economic value of the self‐monitoring intervention through a willingness‐to‐pay (WTP) analysis, whose methodological details and findings are presented in this paper.

## 2 Methods

### 2.1 Overview of the Feasibility Study

The detailed results from the REVERSE study are presented elsewhere [14]. As a prospective, non‐randomized feasibility study, all participants received the self‐monitoring intervention. Patients were asked to monitor their BP on the first three days of each month for 6‐months, taking two readings on each occasion. Each daily second reading, as well as the overall month, were color coded using a traffic light based algorithm (Figure S1) to classify their readings as either good (normotensive), raised (prehypertensive) or high (hypertensive). Monthly readings were submitted to the research team and, if readings were high for two consecutive months, then patients informed their general practice (GP).

Recruitment involved GPs, pharmacies and community providers and was mainly driven by participating GPs. Eighty individuals were enrolled in the study, with 75 starting the intervention. While the recruitment strategy aimed to ensure socioeconomic diversity, most of the participants were White, of high socioeconomic background and well‐educated.

At follow‐up, there was attrition of 17% at 6‐months when the intervention formally stopped, and of 41% at 12‐months when patients had the option of continuing the self‐monitoring should they have wished to do so. Intervention fidelity was high but decreased over time [15]. Importantly, the intervention and study processes were acceptable to both participants and HCPs.

### 2.2 WTP Questionnaire

The WTP analysis was based on a questionnaire developed for this purpose. The questionnaire was designed using the contingent valuation method, a survey‐based approach that estimates the value individuals are willing to pay for a specific good [16]. In our case the “goods” were a BP machine and the additional support and training around BP self‐monitoring (e.g., how to use the BP machine, how to interpret the readings, and what action to take). The development of the questionnaire was informed by a previous version adopted by Kaambwa et al. [17] who explored WTP in self‐monitoring for hypertension.

The questionnaire had 9 questions in total (see Table 2). Question 1 asked if the participants already owned a BP machine and for its cost and other details of the purchase. Question 2 was a multiple‐choice question enquiring whether anyone had previously recommended that the participant should buy a BP machine, with possible choices including HCPs, family, friends, and patient groups. Question 3 was a Likert scale question measuring the relevance of the factors that participants would consider when deciding whether to purchase a BP machine, such as its price, accuracy, ease of use, make or model, or the machine being the one used by the participant's GP.

Question 4 asked how much participants would be willing to pay for a BP machine, providing ranges of market prices for BP machines with increasingly additional features: a basic BP machine that may or may not have a memory; a BP machine with more accuracy and memory; and a more advanced BP machine with memory and the ability to send readings to the participant's healthcare provider. Question 5 was a multiple‐choice question investigating the reasons behind the participants’ WTP amount expressed in Question 4. In a similar fashion, Questions 6 and 7 asked whether participants would be willing pay more for additional support and training around BP self‐monitoring and, where applicable, how much more and why.

Lastly, Questions 8 and 9 enquired about the participants’ household income and whether they received benefits or not.

The patient‐reported data was collected by a researcher at a face‐to‐face or online research clinic during the study 6‐month follow‐up.

### 2.3 Analysis

Using a regression analysis framework, we investigated the direction and magnitude of the associations between the dependent variable (i.e., total WTP amount) and a set of independent variables delineating the demographic and clinical characteristics of the participants. The total WTP amount was calculated by adding the WTP amount for a BP machine (as per Question 4) and the WTP amount for additional support and training around self‐monitoring (as per Question 6). In the initial model specification a set of 20 independent variables was considered comprising: (1) gender (categorical); (2) age in years (continuous); (3) past history of high cholesterol (binary); (4) current ongoing treatment (binary); (5) Body Mass Index (BMI) in kg/m^2^ (continuous); (6) deprivation decile as per the Index of Multiple Deprivation (categorical); change in (7) systolic and (8) diastolic BP in mmHg from baseline to 6 months (both continuous); (9) EuroQol 5‐Dimension 3‐Level (EQ‐5D‐3L) utility score at baseline (continuous); (10) gross annual household income (categorical); reasons behind the WTP amount for (11–15) BP machine and (16–20) additional support and training around BP self‐monitoring (all binary). Given the small sample size of the feasibility study, a selection of variables was deemed necessary. This was conducted using backward stepwise selection with ordinary least squares regressions based on a log‐transformed dependent variable, as the total WTP amount was right‐skewed. The backward stepwise selection gradually removed variables with a *p*‐value higher than 0.2 and added in variables with a *p*‐value lower than 0.1. Gender was kept in throughout given its importance in the model by Kaambwa et al. [17]; no other a priori restrictions were put in place.

The final specification, as selected using backward stepwise selection, was analyzed with generalized linear modelling (GLM) given the expected heteroskedasticity (i.e., variance of residuals was not constant) and non‐normality of the dependent variable (i.e., total WTP amount) [18]. The modified Park test was used to select the appropriate distribution for the dependent variable [19]. Missing data were handled using multiple imputation analysis with different methods applied according to the type of variables affected by missing data (i.e. ordinal or continuous) [20]. The results from the multiple imputation analysis were compared with those from the complete case analysis. Prices’ variables were originally collected in 2023 Great Britain pound (GBP) and then converted into 2024 GBP using an online converter [21]. The analysis was conducted using Stata 17 [22].

## 3 Results

### 3.1 Sample Characteristics

In Table 1 we present the descriptive statistics of the 66 respondents to the WTP questionnaire. Thirty‐nine respondents were female (59.09%) and the median age was 58.50 years (interquartile range, IQR: 52.00 to 65.75 years). Of those respondents stating their ethnicity (*n* = 64), all identified as being White. Most of the respondents lived in areas with lower levels of deprivation (*n* = 48 in deprivation deciles ≥ 6, 72.73%), but 32 respondents (58.18% of those with valid data) had a gross annual household income of less than £50 000 (in 2023 prices, equivalent to just below £52 000 in 2024 prices). Four respondents had a past history of high cholesterol (6.06%) and 12 respondents (18.18%) were undergoing treatment or follow‐up in hospital. The median BMI was 24.83 (IQR: 22.75 to 27.97), with half of the respondents having a healthy weight (BMI between 18.5 and 24.9: *n* = 32, 50.00% of *n* = 64 with valid data). From baseline to 6 months, the mean systolic and diastolic BP of the participants increased by 4.46 mmHg (standard deviation, SD: 12.07 mmHg) and 1.70 mmHg (SD: 9.14 mmHg), respectively (for *n* = 56 valid cases).

**TABLE 1:** Sociodemographic and clinical characteristics of sample (*N* = 66).

Gender	
Females n (%)	39 (59.09)
Age in years	
Median (IQR)	58.50 (52.00 to 65.75)
Ethnicity	
Not reported n (%)	2 (3.03)
White n (%)	64 (100.0)
Index of Multiple Deprivation	
3rd decile n (%)	5 (7.58)
4th decile n (%)	4 (6.06)
5th decile n (%)	9 (13.64)
6th decile n (%)	11 (16.67)
7th decile n (%)	7 (10.61)
8th decile n (%)	8 (12.12)
9th decile n (%)	14 (21.21)
10^th^ decile n (%) [Least deprived]	8 (12.12)
Working status	
Full time n (%)	32 (48.48)
Part time n (%)	11 (16.67)
Not working n (%)	2 (3.03)
Retired n (%)	21 (31.82)
Highest education achieved	
GCSE or equivalent (age 16) n (%)	13 (19.70)
A‐level of equivalent (age 18) n (%)	14 (21.21)
Degree or equivalent n (%)	18 (27.27)
Post‐graduate/ professional qualification n (%)	19 (28.79)
Doctoral n (%)	2 (3.03)
Current BP in mmHg	
Systolic—6 Months	
Missing n (%); Valid cases n (%)	8 (12.12); 58 (87.88)
Median (IQR)	134.00 (124.75 to 142.00)
Systolic—Change from Baseline to 6 Months	
Missing n (%); Valid cases n (%)	10 (15.15); 56 (84.85)
Mean (SD; 95% CI; range)	4.46 (12.07; 1.23 to 7.70; −25.00 to 40.00)
Diastolic—6 Months	
Missing n (%); Valid cases n (%)	8 (12.12); 58 (87.88)
Median (IQR)	82.00 (75.75 to 88.25)
Diastolic—Change from Baseline to 6 Months	
Missing n (%); Valid cases n (%)	10 (15.15); 56 (84.85)
Mean (SD; 95% CI; range)	1.70 (9.14; −0.75 to 4.14; −16.00 to 24.00)
BMI	
Missing n (%); Valid cases n (%)	2 (3.03); 64 (96.97)
Median (IQR)	24.83 (22.75 to 27.97)
Underweight n (%)	1 (1.56)
Healthy weight n (%)	32 (50.00)
Overweight n (%)	22 (34.38)
Obese n (%)	9 (14.06)
BMI—Change from Baseline to 6 Months	
Missing n (%); Valid cases n (%)	3 (4.55); 63 (95.45)
Mean (SD; range)	−0.42 (1.23; −5.08 to 2.11)
Personal clinical history: “Have you ever, or do you currently have, any of the following?”	
High cholesterol n (%)	4 (6.06)
Current ongoing treatment/ follow‐up in hospital? n (%)	12 (18.18)
Hospital admissions n (%)	6 (9.09)

Abbreviations: BMI, body mass index; BP, blood pressure; CI, confidence interval; IQR, interquartile range; SD, standard deviation.

The data from the WTP questionnaire (Table 2) showed that the majority of participants did not previously own a BP machine (*n* = 42, 63.64%) and had not been recommended that they should buy one (*n* = 56, 84.85%). In the scenario where the participants would buy a BP machine, most of them would consider its accuracy, ease of use, and price as the most important factors behind the purchase (for 100%, 95.38%, and 60.00% of respondents, respectively). The median total WTP amount was £41.37 (IQR: £36.20 to £93.09), consisting of the WTP amount for the BP machine (median: £41.37; IQR: £31.03 to £62.06) and the WTP amount for additional support and training around BP self‐monitoring (median: £31.03; IQR: £20.69 to £51.72). Twenty‐four respondents (36.36%) indicated that they would be willing to pay more to receive additional support and training around BP self‐monitoring, but only *n* = 22 of these (91.67%) expressed the related WTP amount. The amounts expressed by the respondents mostly reflected the functions/facilities they would want from a BP machine (*n* = 49, 74.24% of *N* = 66) and the potential benefit from additional support and training around BP self‐monitoring (*n* = 16, 66.67% of *N* = 24).

**TABLE 2:** WTP questionnaire (*N*=66 unless otherwise specified).

Q1. Do you currently own a BP machine (not including the one given to you for this study)?	
No n (%)	42 (63.64)
Q2. Has anyone ever recommended that you buy a BP monitor?	
Not recommended n (%)	56 (84.85)
Q3. What would you consider important if you were buying a BP machine?^a^	
Missing n (%)	1 (1.52)
Price n (%)	39 (60.00)
Accuracy n (%)	65 (100.00)
Ease of use n (%)	62 (95.38)
Make or model n (%)	5 (7.69)
Machine my GP uses n (%)	24 (36.92)
Q4. What is the most you would be willing to pay to buy a BP machine?^b^	
Median (IQR: range), £	41.37 (31.03 to 62.06; 20.69 to 103.44)
Q5. Why would this be the most you would be willing to pay?	
Amount reflects ability to pay n (%)	19 (28.79)
Amount is reasonable value n (%)	33 (50.00)
Amount reflects satisfaction with equipment n (%)	32 (48.48)
Amount reflects potential benefit n (%)	38 (57.58)
Amount reflects machine functions/facilities I would want (%)	49 (74.24)
Other n (%)	13 (19.70)
Q6a. Would you be willing to pay more to receive additional support and training around BP self‐monitoring?	
Yes n (%)	24 (36.36)
Q6b. How much would you be willing to pay for this additional support and training plus the machine?^b^ (*n* = 24^c^)	
Missing n (%)	2 (8.33)
Median (IQR; range), £	31.03; (20.69 to 51.72; 10.34 to 155.16)
Q7. Why would you be willing to pay this? (*n* = 24^c^)	
Amount reflects ability to pay n (%)	8 (33.33)
Amount is reasonable value n (%)	11 (45.83)
Amount reflects satisfaction with equipment n (%)	5 (20.83)
Amount reflects potential benefit n (%)	16 (66.67)
Amount reflects machine functions/facilities I would want (%)	10 (41.67)
Other n (%)	3 (12.50)
What is the most you would be willing to pay for a BP machine plus additional support and training around BP self‐monitoring?^b^ That is, Total WTP amount^d^	
Median (IQR; range), £	41.37 (36.20 to 93.09; 20.69 to 196.53)
Q8. What is the annual income of your household before deducting tax and national insurance?^e^	
Missing n (%)	1 (1.52)
≤ £20 000 n (%)	3 (4.55)
£20 001–£30 000 n (%)	12 (18.18)
£30 001–£40 000 n (%)	10 (15.15)
£40 001–£50 000 n (%)	7 (10.61)
£50 001–£60 000 n (%)	4 (6.06)
£60 001–£70 000 n (%)	4 (6.06)
£70 001–£80 000 n (%)	2 (3.03)
£80 001–£90 000 n (%)	3 (4.55)
£90 001–£100 000 n (%)	5 (7.58)
>£100 000 n (%)	5 (7.58)
Prefer not to answer n (%)	10 (15.15)
Q9. Do you receive benefits?	
No n (%)	62 (93.94)

Abbreviations: BP, blood pressure; GP, General Practitioner; IQR, interquartile range; WTP, willingness to pay.

^a^ Counts and percentages reflect the sum of those who answered Agree or Strongly Agree in relation to the specific factor.

^b^ Prices originally expressed in 2023 GBP and here presented as 2024 GBP. Conversions done on 18‐Mar‐2024 using an online converter tool (https://eppi.ioe.ac.uk/costconversion/).

^c^ Question only applicable to those who answered ‘Yes’ to previous question.

^d^ Calculated as the sum of the WTP amounts for a BP machine and for additional support and training around BP self‐monitoring (see previous questions).

^e^ Any benefits or pensions are included as income. Income groups expressed in 2023 GBP.

### 3.2 GLM Regression

The independent variables included in the final model (following backward stepwise selection) were gender, history of high cholesterol, changes in systolic and diastolic BP from baseline to 6‐months, gross annual household income, and the set of reasons behind the WTP amount for the BP machine (ability to pay; reasonable value; satisfaction; benefit; and machine functions/facilities).

**FIGURE 1:**
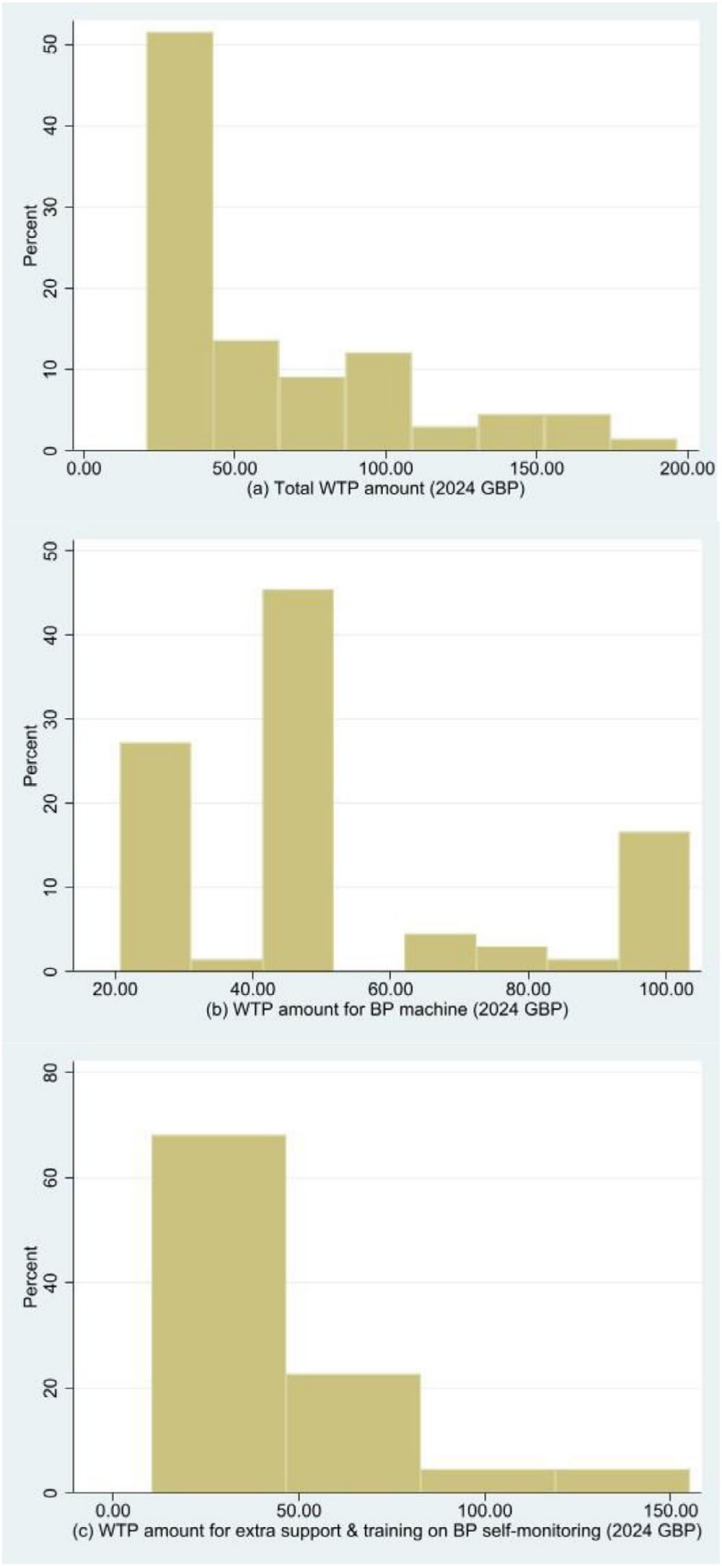
Distributions of (a) total WTP amount and amounts for (b) BP machine and (c) additional support and training around BP self‐monitoring, expressed in 2024 GBP.

The modified Park test suggested that, based on the natural logarithmic coefficient of the predicted total WTP amount equal to 2, Gamma was the most suitable family of distributions for the dependent variable. A log link was employed given the right‐skewed data (as shown in Figure 1) for the dependent variable [17]. In the multiple imputation analysis (Table 3), within the set of independent variables only two specific reasons behind the WTP amount for the BP machine had a statistically significant association with total WTP amount: potential benefits and functions/facilities of the BP machine. In particular, both reasons were associated with an increase in the total WTP amount.

**TABLE 3:** Generalized linear regression model on Total WTP amount^a^ (for BP machine plus additional support and training around BP self‐monitoring), with Gamma distribution^b^ and log link—Multiple imputation analysis.

Dependent variable: Total WTP amount^a^ (*N* = 66)				
Independent variable^c^	Coefficient	SE	*p*‐value	95% CI
Gender				
Male	Reference category			
Female	−0.08	0.15	0.61	−0.38 to 0.22
High cholesterol				
No	Reference category			
Yes	0.45	0.32	0.16	−0.18 to 1.08
Change in systolic BP from Baseline to 6 Months	−0.01	0.01	0.16	−0.03 to 0.00
Change in diastolic BP from Baseline to 6 Months	−0.01	0.01	0.17	−0.04 to 0.01
Gross annual household income^d^				
≤ £20 000 n (%)	Reference category			
£20 001–£30 000 n (%)	−0.17	0.39	0.67	−0.93 to 0.60
£30 001–£40 000 n (%)	−0.23	0.33	0.49	−0.88 to 0.42
£40 001–£50 000 n (%)	−0.21	0.36	0.56	−0.93 to 0.50
£50 001–£60 000 n (%)	−0.16	0.47	0.73	−1.09 to 0.76
£60 001–£70 000 n (%)	−0.33	0.45	0.46	−1.22 to 0.56
£70 001–£80 000 n (%)	0.53	0.52	0.31	−0.49 to 1.56
£80 001–£90 000 n (%)	0.56	0.53	0.29	−0.49 to 1.61
£90 001–£100 000 n (%)	−0.08	0.40	0.84	−0.87 to 0.71
> £100 000 n (%)	−0.16	0.40	0.69	−0.94 to 0.63
WTP amount for BP machine reflecting ability to pay				
No	Reference category			
Yes	−0.29	0.17	0.080	−0.61 to 0.03
WTP amount for BP machine being reasonable value				
No	Reference category			
Yes	−0.13	0.15	0.37	−0.42 to 0.16
WTP amount for BP machine reflecting satisfaction with equipment				
No	Reference category			
Yes	−0.09	0.15	0.53	−0.39 to 0.20
WTP amount for BP machine reflecting potential benefit				
No	Reference category			
**Yes**	**0.52**	**0.17**	**0.002**	**0.19 to 0.85**
WTP amount for BP machine reflecting machine functions/facilities I would want				
No	Reference category			
**Yes**	**0.38**	**0.18**	**0.040**	**0.02 to 0.74**
Constant	3.90	0.31	<0.001	3.30 to 4.51

Abbreviations: BP: blood pressure. CI: confidence interval. SE: standard error. WTP: willingness‐to‐pay. **In bold**: statistically significant results (at the 5% significance level).

^a^ Prices originally expressed in 2023 GBP and here presented as 2024 GBP. Conversions done on 18‐Mar‐2024 using an online converter tool (https://eppI.ioe.ac.uk/costconversion/default.aspx).

^b^ Gamma distribution selected by using the modified Park test.

^c^ Independent variables selected using backward stepwise selection.

^d^ Income groups expressed in 2023 GBP.

Lastly, the results from the multiple imputation analysis differed from those of the complete case analysis (based on *N* = 46 observations), where a greater number of variables had a statistically significant association with total WTP amount (Table S1). This difference attests the non‐negligible impact of missing data which affected the variables related to the changes in systolic and diastolic BP (both had 15.15% of missing data) and gross annual household income (16.67% of missing data).

## 4 Discussion

### 4.1 Summary

The results from the WTP questionnaire showed that over one‐third of participants in this feasibility study were willing to pay more to receive additional support and training around BP self‐monitoring. The range of WTP amounts for this extra support and training was quite large (£20.69 to £51.72), attesting how the perceptions around its value varied considerably in our sample. Analysis of total WTP for the BP machine, including additional support and training, showed that only two variables, both related to the device's benefits and features, were statistically significant. This suggests that participants’ WTP was shaped by perceptions of the BP machine's capabilities and the value these features could provide. This underlines how, at least in our sample, it is the machine's characteristics that drive the value seen by the participants. Therefore, based on this finding, the range of capabilities and features of the BP machine, as perceived by the participants, represents an aspect to be prioritized when it comes to design self‐monitoring interventions for prehypertensive people.

Conversely, variables reflecting reasons for the WTP amounts related to additional training and support for BP self‐monitoring could not be included in the final model specification due to multicollinearity (i.e., several independent variables were related to each other and providing similar information) and the small number of observations (*n* = 24). Associations between sociodemographic and clinical variables and the total WTP amount were not statistically significant. However, we recommend testing these findings in larger, more diverse studies to evaluate the potential role that training and support, and sociodemographic and clinical characteristics may play in driving the WTP values.

### 4.2 Strength and Limitations

The main strength of this study lies in it being one of the first to investigate the economic value of a self‐monitoring intervention for people in the prehypertensive BP range. However, some limitations affect our study. First, the small study sample restricts any considerations around the generalizability of the findings. This small sample size impacted on the specification of the regression model, which was formalized through a careful selection of the most relevant variables in order to preserve an adequate number of degrees of freedom (i.e., variability). Second, and linked to the first limitation, the volume of missing data affecting some of the variables (changes in systolic and diastolic BP, and gross annual household income) could also have hampered the generalizability of the results. To address this, multiple imputation was applied to those variables and resulted in a non‐negligible difference in the results compared with those from the complete case analysis. Third, the representativeness of the sample is limited given that all participants identified their ethnicity as White, with most living in areas with low levels of deprivation and having low notable clinical history (i.e., many participants did not have an history of high cholesterol, or had ongoing treatments, and were in a healthy weight range). Fourth, only a sub set of participants (*n* = 24) were willing to pay more for additional support and training around BP self‐monitoring, meaning that the associated role in shaping the economic value of self‐monitoring could not be analyzed in depth.

### 4.3 Comparison With Existing Literature

Our findings partially contrast with those of the WTP analysis by Kaambwa et al. [17] which had similar analytical grounds to ours but focused on 393 people with hypertension. Kaambwa et al. [17] found that WTP amounts were associated with gender (i.e. being male), having a midrange household income and satisfaction with BP machine. However, similar to our results, Kaambwa et al. [17] found that changes in BP were not associated with WTP amounts. Comparisons with other papers are challenging as ours appears to be the first study that investigated the economic value of a self‐monitoring intervention specifically for people with BP in the prehypertensive range. The lack of similar literature appears to be substantiated by our (unpublished) systematic review which did not find any economic evaluations on non‐pharmacological interventions for people with prehypertension [23].

### 4.4 Implications for Research

Our study appears to be the first to attempt to place an economic value to a self‐monitoring intervention specifically delivered to people with BP in the prehypertensive range. As our analysis was performed alongside a single‐arm feasibility study, any interpretation of the findings will need to be made with caution. Larger studies will need to corroborate or contrast our findings, accounting also for a more representative sample, with a greater range of people living in areas with different levels of deprivation, and with different clinical profiles.

## Funding

The REVERSE study is funded by the National Institute for Health and Care Research ‐ Research for Patient Benefit Programme (NIHR RfPB) (NIHR201028). The views expressed are those of the authors and not necessarily those of the NIHR or the Department of Health and Social Care. VB, AC and CLW were funded by the National Institute for Health and Care Research Applied Research Collaboration North West Coast (NIHR ARC NWC). The views expressed in this publication are those of the authors and not necessarily those of the National Institute for Health and Care Research or the Department of Health and Social Care.

## Ethics Statement

The study is registered (ISRCTN13649483) and favorable ethical opinion was received from London–Fulham NHS Research Ethics Committee (REF: 22/PR/0108) and the University of Lancashire Health Ethics Review Panel (HEALTH 0299).

## Consent

No written consent has been obtained from the patients as there is no patient identifiable data included in this article.

## Conflicts of Interest

The Authors declare that there is no conflict of interest.

## REVERSE Study Group

Bray E.P., Hives L., Georgiou R., Benedetto V., Heyworth P., Doherty P., Williams N., Rutter P., Spencer J., Clegg A., Watkins C.L.

## Supporting information




**Figure S1**. BP coding algorithm.
**Table S1**. Generalized linear regression model on Total WTP amount^a^ (for BP machine plus additional support and training around BP self‐monitoring), with Gamma distribution^b^ and log link—Complete‐case analysis.

## Data Availability

The data used in this study is confidential and cannot be shared.
