# Supplementary material for: Willingness to Pay for Blood Pressure Self‐Monitoring in People With Prehypertension
Source: J Clin Hypertens (Greenwich). 2026 Apr 11;28(4):e70247. doi: 10.1111/jch.70247 (PMC13069489; doi:10.1111/jch.70247)
Supplement: Supplementary file 1 — Figure S1. BP coding algorithm. Table S1. Generalized linear regression model on Total WTP amounta (for BP machine plus additional support and training around BP self‐monitoring), with Gamma distributionb and log link—Complete‐case analysis. [file JCH-28-e70247-s001.docx]

**Title:**

**Willingness to pay for blood pressure self-monitoring in people with pre-hypertension**

# Supplementary data

## Figure S1. Blood pressure coding algorithm.
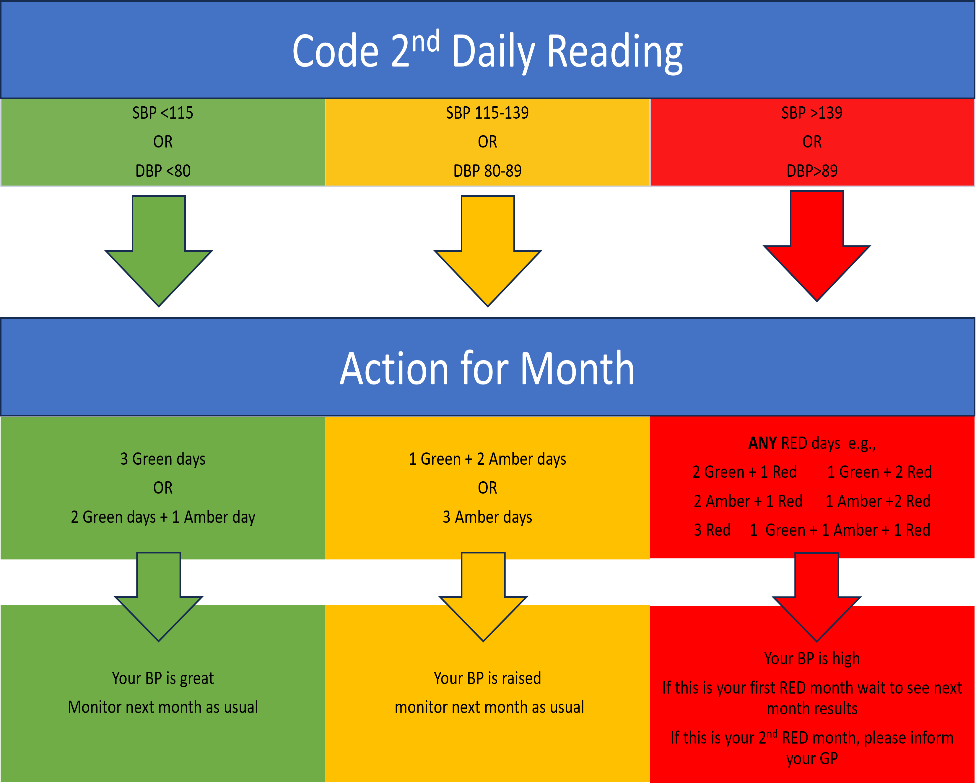


Note: based on standard clinical guidelines and reduced by 5/5 mmHg as recommended in the literature for home readings.

## Table S1. Generalised linear regression model on Total WTP amount^a^ (for blood pressure machine plus additional support and training around BP self-monitoring), with Gamma distribution^b^ and log link – Complete-case analysis

| **Dependent variable: Total WTP amount^a^**  **(n=46 complete cases)** | | | | |
| --- | --- | --- | --- | --- |
| **Independent variable^c^** | **Coefficient** | **SE** | **p-value** | **95% CI** |
| Gender | | | | |
| *Male* | *Reference category* | | | |
| *Female* | 0.25 | 0.15 | 0.090 | -0.04 to 0.54 |
| High cholesterol | | | | |
| *No* | *Reference category* | | | |
| ***Yes*** | **0.77** | **0.29** | **0.009** | **0.19 to 1.34** |
| **Change in systolic BP from Baseline to 6 Months** | **-0.03** | **0.01** | **<0.001** | **-0.04 to -0.01** |
| Change in diastolic BP from Baseline to 6 Months | -0.02 | 0.01 | 0.056 | -0.03 to 0.00 |
| Gross annual household income^d^ | | | | |
| *<=£20,000 n (%)* | *Reference category* | | | |
| ***£20,001 to £30,000 n (%)*** | **-0.63** | **0.30** | **0.038** | **-1.22 to -0.03** |
| *£30,001 to £40,000 n (%)* | -0.39 | 0.27 | 0.16 | -0.92 to 0.15 |
| ***£40,001 to £50,000 n (%)*** | **-0.59** | **0.28** | **0.034** | **-1.14 to -0.04** |
| *£50,001 to £60,000 n (%)* | -0.56 | 0.37 | 0.14 | -1.29 to 0.18 |
| *£60,001 to £70,000 n (%)* | -0.52 | 0.34 | 0.13 | -1.19 to 0.16 |
| *£70,001 to £80,000 n (%)* | 0.51 | 0.38 | 0.18 | -0.23 to 1.25 |
| *£80,001 to £90,000 n (%)* | 0.39 | 0.38 | 0.31 | -0.36 to 1.15 |
| *£90,001 to £100,000 n (%)* | -0.37 | 0.31 | 0.23 | -0.97 to 0.23 |
| *>£100,000 n (%)* | -0.63 | 0.32 | 0.052 | -1.26 to 0.00 |
| **WTP amount for BP machine reflecting ability to pay** | | | | |
| *No* | *Reference category* | | | |
| ***Yes*** | **-0.32** | **0.15** | **0.028** | **-0.61 to -0.03** |
| **WTP amount for BP machine being reasonable value** | | | | |
| *No* | *Reference category* | | | |
| ***Yes*** | **-0.29** | **0.14** | **0.035** | **-0.55 to -0.02** |
| WTP amount for BP machine reflecting satisfaction with equipment | | | | |
| *No* | *Reference category* | | | |
| *Yes* | -0.19 | 0.13 | 0.149 | -0.46 to 0.07 |
| **WTP amount for BP machine reflecting potential benefit** | | | | |
| *No* | *Reference category* | | | |
| ***Yes*** | **0.36** | **0.16** | **0.021** | **0.05 to 0.67** |
| **WTP amount for BP machine reflecting machine functions/facilities I would want** | | | | |
| *No* | *Reference category* | | | |
| ***Yes*** | **0.46** | **0.16** | **0.005** | **0.14 to 0.78** |
| Constant | 4.27 | 0.25 | <0.001 | 3.78 to 4.77 |

BP: blood pressure. CI: confidence interval. SE: standard error. WTP: willingness-to-pay. **In bold**: statistically significant results (at the 5% significance level).

^a^ Prices originally expressed in 2023 GBP and here presented as 2024 GBP. Conversions done on 18-Mar-2024 using an online converter tool (<https://eppI.ioe.ac.uk/costconversion/default.aspx>).

^b^ Gamma distribution selected by using the modified Park test.

^c^ Independent variables selected using backward stepwise selection.

^d^ Income groups expressed in 2023 GBP.
